# Complex Relationships between Milking-Induced Changes in Teat Structures and Their Pre-Milking Dimensions in Holstein Cows

**DOI:** 10.3390/ani13061085

**Published:** 2023-03-17

**Authors:** Matúš Gašparík, Iveta Szencziová, Jaromír Ducháček, Eva Tóthová Tarová, Luděk Stádník, Melinda Nagy, Lucie Kejdová Rysová, Marek Vrhel, Veronika Legarová

**Affiliations:** 1 Department of Animal Science, Faculty of Agrobiology, Food and Natural Resources, Czech University of Life Sciences Prague, Kamýcká 129, 165 00 Prague, Czech Republic; duchacek@af.czu.cz (J.D.);; 2 Department of Biology, Faculty of Education, J. Selye University in Komárno, Bratislavská cesta 3322, 945 01 Komárno, Slovakia; szencziovai@ujs.sk (I.S.);; 3 Department of Food Science, Faculty of Agrobiology, Food and Natural Resources, Czech University of Life Sciences Prague, Kamýcká 129, 165 00 Prague, Czech Republic

**Keywords:** dairy cow, teat canal, teat morphology, teat wall, ultrasonography

## Abstract

**Simple Summary:**

Multiple studies have investigated the relationship of teat morphology, and udder health and milkability. Nevertheless, it is crucial to examine how the dimensions of teat structures influence teat tissue changes during milking. The short-term changes in teat structures that result from machine milking are yet to be completely understood, and more studies are needed to fully understand their complexity and potential health risks to animals. We found that milking-induced changes in teat structures depended on their pre-milking size, probably because their dimensions allowed for certain fluid mechanics inside the teat after the forces were applied within the liner of specific dimensions. Furthermore, the dimensions of any teat structure affected changes in multiple other structures. We also observed that some teat dimensions showed a better reaction to milking in relation to milking-induced teat tissue changes. The results of this study could create a better understanding of milking-induced changes in teat tissue. Our findings may help create a detailed computer model for teat reaction to milking, which could be used to improve the development of milking equipment.

**Abstract:**

The study aimed to explore the relationship between teat structure dimensions and their short-term reaction to milking, to find the optimal dimensions of teat structures in relation to milking-induced teat tissue changes. Teat structures (teat length, canal length, thickness at barrel and apex, wall and cistern width) were measured by ultrasonography before and after milking for 38 Holstein cows at the beginning, middle, and end of lactation. We found that milking-induced changes in teat structures significantly depended on their pre-milking size. Furthermore, we observed that some teat structures and their changes were interconnected, and some did not affect each other. For example, changes in the barrel thickness and cistern width were affected by all structures, while the canal and apex did not influence each other. We deduced that more favorable changes were observed for teats of medium length, medium barrel and apex thickness, with teat canals of medium length, but with wider cisterns and thinner walls. The results of this study may help improve research in the area of milking-induced changes in teat morphology. Our findings could help understand potential health risks to animals in relation to teat morphology, milking equipment, and machine settings.

## 1. Introduction

It is generally agreed that machine milking induces changes (long-, medium-, and short-term) in teat canal integrity and teat tissue pliability [1,2,3]. Short-term changes are defined as tissue responses to a single milking event and can be distinguished into congestion (intravascular accumulation of fluids) or edema (extravascular accumulation of fluids) [4]. These alterations in the teat circulatory system have been associated with increased susceptibility to new intramammary infection [5]. Sufficient blood circulation in the teat tissue is paramount for the defense mechanisms of the teat against mastitis pathogens [6].

Consequently, several associated factors have been reported that help explain the variability of machine-milking-induced short-term changes in teat tissue condition, such as fluctuations of teat end vacuum [7], mouthpiece chamber vacuum [2], milking liner design [8], pulsation ratio and rate [9], and milking duration [4]. Another source of variability could be differences in teat anatomy, which may influence the susceptibility to short-term changes [10]. Teat morphology differs between breeds [11,12], cows, and even quarters within cows [13].

Cows with higher udders, deeper central ligaments, tighter attachments, and centrally placed teats of medium length are the most desirable from the udder conformation standpoint [14]. However, internal teat traits are not evaluated in practice. Studies found a relationship between barrel thickness, apex thickness [15], and teat canal [11,16] and milkability or udder health. Milking-induced changes in these structures have also proven to be important. Zwertvaegher et al. [17] observed that the teats which thickened during milking had a higher somatic cell count than the teats that decreased in barrel diameter. Furthermore, changes in teat apex diameter by more than 5% increased the colonization of the teat canal by pathogens [18]. A similar threshold of ±5% change in apex diameter was suggested by Hamann and Mein [9], to evaluate the effectiveness of pulsation in relation to the type of liner and vacuum level. Wieland et al. [19] suggested that differences in short-term changes can be attributed mainly to the variation in anatomical dimensions and structures among teats with different teat-end shapes, resulting in differences in milk flow and liner fit.

To the best of our knowledge, the short-term changes in teat structures that result from machine milking still need to be fully understood, mainly in the context of the relation of the optimal response of individual teat structures to milking, and the extent to which these changes are affected by the dimensions of teat structures. More studies are needed to understand their complexity and potential health risks to animals. Many studies have focused on the influence of teat structures on udder health and milkability, but it is also essential to investigate to what extent individual structures influence teat tissue changes. The study aimed to explore the relationship between the dimensions of the teat structures and their short-term reaction to milking, to find the optimal dimensions of teat structures in relation to milking-induced teat tissue changes.

## 2. Materials and Methods

The study was conducted at a commercial dairy farm with Holstein cows in the Central Bohemian region of the Czech Republic. The cows were housed in a modern stable with free-stall housing and recycled manure solids as bedding. One electric cow brush was available in each section of the stable. The cows were milked twice a day in a herringbone parlor (2 × 12). The critical milk flow for the automatic detachment system was set to 0.5 kg min^−1^. Pulsation was set to a 60:40 ratio, with 55 pulses per minute. The vacuum level was set to 42 kPa. The teat liners had a triangular design, with a 22.5 mm orifice diameter (Milkrite ImpulseAir IP10U ventilated; Milkrite InterPuls; Johnson Creek, WI, USA).

The morphological dimensions of the teat structures were measured at the beginning (3–17 days in milk), middle (149–165 days in milk), and end of lactation (293–314 days in milk) for 38 Holstein cows (first lactation *n* = 12; second lactation *n* = 12; third lactation *n* = 7; fourth lactation *n* = 7). All teats of cows in the test were evaluated once during each period. All cows that calved on this farm during July and August, and were not culled during their lactation, were included in the experiment. Teat measurements took place immediately before and after the evening milking by a trained researcher. Teat length (LENGTH) was measured by a caliper. Teat barrel thickness (BARREL), teat cistern width (CISTERN), teat wall thickness (WALL), teat apex thickness (APEX), and teat canal length (CANAL) were measured by ultrasonography (Aloka SSD500 machine with 7.5 MHz linear probe; Hitachi Aloka Medical; Tokyo, Japan), with the warm water bath method [1]. We chose to use the water bath method over the direct contact method, as it provides better images of the whole teat without any possible deformations, due to a lack of contact between the probe and the teat or udder tissue. Water in the cup was changed after each teat to prevent cross-contamination by mastitis pathogens.

**Figure 1:**
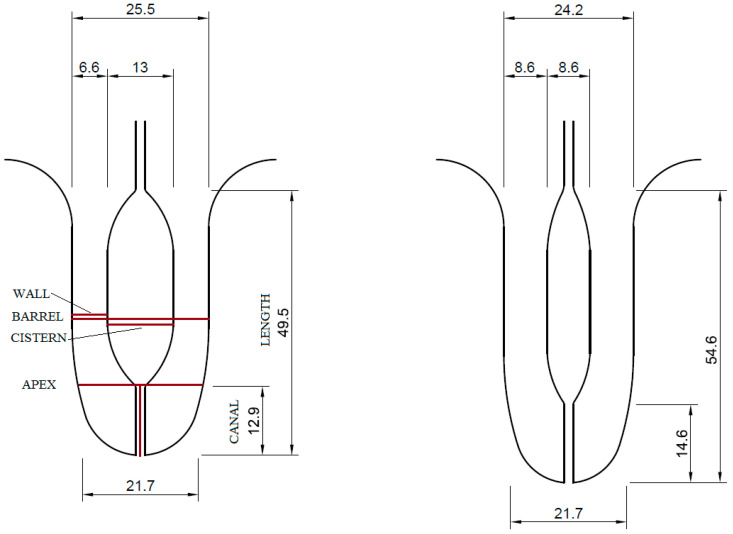
An illustration of the average size of the teat structures before (**left**) and after (**right**) milking in mm, with the location of the measurements. Only the teat wall further from the probe was measured, and sometimes it was measured at a different angle than barrel thickness, which, together with the standard deviation, caused a slight discrepancy between barrel thickness and the thickness of wall + cistern + wall.

Collected ultrasonographic video records were processed in the NIS-Elements AR 3.2 (Nikon Corp.; Tokyo, Japan). In total, 912 videos were evaluated (38 cows × 4 teats × 2 before/after milking × 3 lactation stages). Teat structures were measured at standard locations, as in the study by Neijenhuis et al. [1] or Wieland et al. [10]. Therefore, BARREL, CISTERN, and WALL were measured at 10 mm from Furstenberg’s rosette, while APEX was measured at Furstenberg’s rosette. CANAL was measured from Furstenberg’s rosette to the end of the teat (Figure 1). Milking-induced changes of LENGTH (LENGTH%), BARREL (BARREL%), CISTERN (CISTERN%), WALL (WALL%), APEX (APEX%), and CANAL (CANAL%) were calculated as:

(1) $$\mathrm{Milking}-\mathrm{induced}\;\mathrm{change}\;\%=\left[\left(\mathrm{postmilking}\;\mathrm{value}-\mathrm{premilking}\;\mathrm{value}\right)÷\mathrm{premilking}\;\mathrm{value}\right]\times 100$$

The dataset file is available in a publicly accessible repository [20]. Complementary data about the cows in the test were taken from farm evidence (lactation number, days in milk) and in-line real-time milk analyzers (milk yield, protein, lactose, and fat content; Afilab with software Afifarm 4.1; Afimilk; Afikim; Isreal).

Program SAS 9.3. (SAS Institute Inc.; Cary, NC, USA) was used for statistical evaluation. The UNIVARIATE procedure was used for basic statistics. The FREQ procedure was used to calculate the frequencies for pre-milking dimensions of teat structures. The effect of pre-milking dimensions of teat structures (experimental unit) on their milking-induced changes (dependent variables) was evaluated in the MIXED procedure. The STEPWISE method in the REQ procedure was used to select a suitable model for evaluating the selected parameters. Effects with significant influence on teat morphology and milking-induced changes, such as lactation number, lactation stage, and teat position, were included in the model equation [20]. The best model for evaluation was selected by the values of the Akaike information criterion. The model equation included the random effect of the animal, fixed effects of the lactation number (primiparous, *n* = 144; multiparous, *n* = 312), lactation stage (early, *n* = 152; mid, *n* = 152; late, *n* = 152), and teat position (front, *n* = 228; rear *n* = 228). Lastly, the morphology effect of individual teat structure before milking was added to the equation. The fixed effects of teat morphology before milking were individually evaluated and were divided based on frequency into equal groups: LENGTH (<43.5 mm—small, *n* = 136; 43.5–52.5 mm—medium, *n* = 157; 52.5< mm—long, *n* = 163); BARREL (<24.2 mm, *n* = 151; 24.2–26.5 mm, *n* = 149; 26.5< mm, *n* = 156), CISTERN (<11 mm, *n* = 152; 11–14.5 mm, *n* = 151; 15< mm, *n* = 153), WALL (<5.7 mm, *n* = 151; 5.7–7 mm, *n* = 152; 7< mm, *n* = 153), APEX (<21 mm, *n* = 152; 21–22.5 mm, *n* = 151; 22.5< mm, *n* =153), and CANAL (<11.8 mm, *n* = 152; 11.8–14 mm, *n* = 151; 14< mm, *n* = 153).

The Tukey–Kramer method was used to evaluate differences in the least square means. Significance level *p* < 0.05 was used to evaluate statistical significance.

## 3. Results

### 3.1. Basic Statistics

The animals in our study were Holstein cows, with an average daily milk yield of 31.56 kg and a milking time of 6.94 min during the monitored lactation. Milk had 3.57% protein, 4.0% fat, and 4.98% lactose. The average sizes of the teat structures before and after milking are presented in Figure 1.

Teats were prolonged during milking by 10.38% on average, and BARREL was reduced on average by 5.18% during milking. CISTERN and WALL changed in the opposite directions during milking, when the average WALL% increased by 29.92%, and the average CISTERN% decreased by 33.79%. We only observed a small average APEX%, when it was only slightly reduced in thickness during milking by 0.20%. Lastly, the average CANAL% was 13.4%.

### 3.2. Significance of Effects in the Model Equation

We used six model equations for the evaluation, one for each evaluated structure. The effect of teat structure dimensions on milking-induced changes was almost always significant, except for a few cases: LENGTH% was not affected by WALL (*p* = 0.47–0.94), APEX (*p* = 0.33–0.9), and CANAL (*p* = 0.38–0.94); WALL% was not affected by APEX (*p* = 0.31–0.94); APEX% was not affected by LENGTH (*p* = 0.57–0.94) and CANAL (*p* = 0.38–0.78); and CANAL% was not affected by APEX (*p* = 0.93–0.99).

The effect of lactation number, lactation stage, and teat position was less significant compared to teat structure dimensions, although still significant in most cases. The detailed significance of the effects in the model equation is presented in the supplementary file (Supplemental Tables S1–S6) [20].

#### 3.2.1. Effect of Lactation Number, Lactation Stage, and Teat Position

In this study, we wanted to focus solely on the effect of teat structure dimensions; however, these critical effects had to be considered (Table 1). Cows at a second and higher lactation showed a lower milking-induced change of teat structures compared to cows at a first lactation (*p* < 0.05), with the exception of BARREL% and APEX%, which were similar.

The effect of the lactation stage was also not significant for BARREL% and APEX%; however, we observed the tendency for a lower change in BARREL% in the later stages of lactation. LENGTH% was affected by the lactation stage when we observed lower LENGTH% at the beginning of the lactation compared to the other two stages, which were similar (*p* < 0.05, Table 1). A difference (*p* < 0.05) between the beginning of lactation and the other two stages was also observed for CISTERN%, WALL%, and CANAL%. Furthermore, we found differences between the front and rear teats in LENGTH%, WALL%, and APEX% (*p* < 0.05, Table 1). Higher milking-induced changes for LENGTH% and WALL% were observed for rear teats, while APEX% was negative for rear teats and positive for front teats. The results for the effects of lactation number, lactation stage, and teat position were mostly in accordance with previous studies, and are further commented on and discussed in the supplementary file [20].

#### 3.2.2. Effect of Teat Length on Milking-Induced Changes

LENGTH significantly affected changes in all teat structures during milking, except the APEX% (Table 2). Small teats prolonged in LENGTH% considerably more than long teats (*p* < 0.05; Table 2). BARREL% was reduced by 3.75 ± 0.86% for short teats and 6.97 ± 0.99% for long teats (*p* < 0.05). Teats with the lowest change at BARREL% had the highest change in LENGTH% and vice versa. Teats of medium LENGTH achieved middle values for BARREL% and LENGTH%, although they achieved the highest milking-induced change of CISTERN%, WALL%, and CANAL%. These differences were not significant between medium and long teats (*p* > 0.05); however, changes in these structures for medium LENGTH were greater than for small LENGTH (*p* < 0.05; Table 2).

#### 3.2.3. Effect of Teat Thickness at the Barrel on Milking-Induced Changes

BARREL influenced the reaction of all teat structures to milking (*p* < 0.05; Table 2). The thick BARREL was reduced the most during milking (by 10.82 ± 0.66%), while the thin BARREL slightly thickened (0.95 ± 0.67%; *p* < 0.05). We also observed the highest change of the thick BARREL for CISTERN%, WALL%, and CANAL%. These differences were significant compared to thin BARREL (*p* < 0.05). Thick BARREL prolonged in LENGTH% less than thin BARREL (*p* < 0.05; Table 2). In contrast to LENGTH, medium BARREL showed middle values for change of all teat structures; however, the differences were significant for other BARREL groups only for BARREL%, CISTERN%, and APEX%. The thin BARREL thickened during milking simultaneously at BARREL% and APEX%, while their WALL% was the lowest (Table 2).

#### 3.2.4. Effect of Teat Thickness at the Apex on Milking-Induced Changes

APEX affected milking-induced changes for BARREL%, CISTERN%, and APEX% (*p* < 0.05; Table 2). We observed approximately a 10% reduction in BARREL% and a 5% reduction in APEX% for teats with a thick APEX (*p* < 0.05). On the other hand, teats with a thin APEX significantly thickened at APEX% (*p* < 0.05; Table 2). CISTERN% reduction was 29.8 ± 2.35% for teats with a thin APEX, while CISTERN% for a medium and thick APEX decreased by around 40% during milking.

#### 3.2.5. Effect of Teat Cistern Width on Milking-Induced Changes

CISTERN affected milking-induced changes in all monitored teat structures (*p* < 0.05; Table 3). Teats with a narrow CISTERN prolonged in LENGTH% more than those with a wide CISTERN (*p* < 0.05). In addition, teats with a narrow CISTERN showed a slight increase in BARREL% during milking, while a medium and wide CISTERN showed a reduction in BARREL% (*p* < 0.05). Moreover, if we closely look at the interplay between BARREL%, CISTERN%, and WALL%, we can see that a decrease (*p* < 0.05) of BARREL% for the widest CISTERN was caused by a high reduction in CISTERN%, while the WALL% of these teats thickened considerably (*p* < 0.05; Table 3). We observed only −17.06 ± 2.47% CISTERN% for teats with a narrow CISTERN, while the WALL% of these teats thickened only by 13.49 ± 2.92%. We found the most favorable APEX% for teats with a medium CISTERN, which maintained APEX dimensions similar to ones before milking. APEX% for a narrow CISTERN was the highest even though they showed the lowest increase in WALL%. The teats with a wide CISTERN had very high CANAL%, while CANAL% for a narrow CISTERN prolonged only slightly (*p* < 0.05; Table 3).

#### 3.2.6. Effect of Teat Wall Thickness on Milking-Induced Changes

All of the WALL groups prolonged in LENGTH% similarly during milking. The results for the thick WALL had a similar trend as results for the narrow CISTERN (Table 3). Teats with a thin WALL showed the highest decrease in BARREL% and CISTERN%, while having the highest increase in WALL% and CANAL%. The APEX% of teats with a thick WALL thickened during milking compared to the medium WALL (*p* < 0.05) and thin WALL (*p* < 0.05), which were reduced. However, differences between the groups were not as high as those of CISTERN evaluation, and stayed closer to 0% (Table 3).

#### 3.2.7. Effect of Teat Canal Length on Milking-Induced Changes

Results showed that a short CANAL caused the greatest increase in WALL%, while BARREL% and CISTERN% showed the highest reductions compared to a medium and long CANAL (*p* < 0.05; Table 3). Milking-induced changes of significantly affected teat structures (Table 3) were decreased for medium CANAL and were even lower for teats with a long CANAL. The most extreme difference was observed for CANAL% when the short CANAL was prolonged by 35.41 ± 2.01% and long CANAL only by 1.62 ± 1.57% (*p* < 0.05).

## 4. Discussion

As noticeable from the results, the relations between teat structures and their interaction during milking were complex and significantly affected each other. The dimensions of the teat liner greatly influence the reaction of the teat to milking [8]. The liner encapsulates the teat in physical space and creates a barrier that limits or promotes the expansion of teat tissue. We observed this relationship for a thick BARREL and thick APEX, which reduced in thickness during milking, while a thin BARREL and thin APEX thickened. We can also see these physical limitations in the interplay between the dimensions of the WALL and CISTERN, whereby the CISTERN% reduction was balanced out by a similar increase in WALL% in all cases. Neijenhuis et al. [1] also found that the WALL% and CISTERN% change in opposite directions during milking. Considering the variation in the teat size among breeds, individual animals within the same breed [12], and even between quarters of the same animal [13], the different degrees of interaction between the teat and the liner cannot be avoided [21]. It has been shown that besides vacuum levels, the predominant reason for teat tissue thickening and longitudinal stretching was a too-wide liner bore relative to the teat size [8,22]. We noticed this effect on a thin BARREL and short LENGTH. However, it did not cause greater changes in the inner structures. On the contrary, we observed the lowest CISTERN%, WALL%, and CANAL% for these teats. Furthermore, in this study, a three-sided concave liner was used, which was less damaging to the teat compared to a standard round design, because the forces could be spread out over the larger area of the teat [23]. The extent of milking-induced changes might have been different if a standard round non-ventilated liner had been used.

Besides the liner, forces applied to the teat during milking play a substantial role in affecting milking-induced changes in the dimensions of the teat. Vacuum-induced forces applied during machine milking create stress on the teat, which can evoke mechanical and circulatory impairment of its tissue [6]. Teats are massaged by pulsation to limit the development of congestion and edema in the teat tissues [24]. We observed the highest accumulation of fluids in teats with a thin BARREL or narrow CISTERN, which simultaneously thickened at the BARREL% and the APEX%. This congestion might have decreased the pliability of teat tissue, which might be the reason for the decrease in milking-induced changes of internal teat structures. Lower changes in inner structures probably do not mean a better reaction to milking. We usually observed low milking-induced changes together with dimensions, or short-term changes, that were found to have negative implications for milking efficiency or udder health by previous studies, for example, a positive BARREL% [17], highly positive APEX% [18], short LENGTH and thin BARREL [25]. On the other hand, structures with negative implications, such as a thick APEX [15], showed high milking-induced changes. However, they occurred together with undesirable APEX% [9], while milking-induced changes for LENGTH%, WALL%, and CANAL% were not significant.

Although the same forces were applied to the identical liners in our study, certain dimensions showed more favorable teat tissue response. These results agree with Wieland et al. [10], who also suggested that individual udder or teat characteristics likely account for the greatest variability in short-term changes. Milking-induced changes in teat structures depended on their morphology, probably because their dimensions allow for certain fluid mechanics inside the teat after the forces are applied within the liner of specific dimensions. Furthermore, some of the teat structures and their changes were interconnected (e.g., CISTERN and BARREL to all structures), and some did not affect each other (e.g., APEX and CANAL).

An interesting dynamic was observed for teat length. Short LENGTH prolonged the most, with the lowest reduction in BARREL%, while long LENGTH increased in LENGTH% the least, with the highest reduction in BARREL%. Therefore, the teat volume increased for short teats after milking, while the total volume might have been reduced for long teats. Differences in penetration depth of the milking liner and discrepancies in teat diameter could lead to variations in the seal between the teat and barrel of the milking liner [10]. As the teat expands to fill the liner cavity, regardless of the barrel width, wide bore liners may also be accompanied by an increase in the mouthpiece chamber vacuum [26], which is also consistent with the findings of Borkhus and Rønningen [25]. They recorded a higher mouthpiece chamber vacuum in the thin and short teats, which is often associated with congestion of teat tissue [2].

Studies suggest that increases in BARREL% during milking could be problematic in relation to edema, udder health, and fluid dynamics [17]. Our study observed the highest decrease in BARREL% when the WALL% thickened severely. Furthermore, the relationship between BARREL% and WALL% was mostly indirectly proportional. Hamann et al. [6] suggested that a decreased blood flow period after milking could be associated with increased teat thickness. Therefore, an increase in WALL% could allow for improved circulation of fluids within the teat, resulting in an overall reduction of BARREL%. Wieland et al. [27] found that teat blood circulation increased after pre-milking teat stimulation and decreased after machine milking. However, the after-milking value was still higher than before stimulation. Authors hypothesize that this alteration was due to vacuum-induced mechanical forces during machine milking, evoking circulatory impairment of the teat. We measured the WALL before stimulation, and after milking it thickened, depending on the group, by 13% to 51%, while Neijenhuis et al. [1] observed a 34% change, and Paulrud et al. [5] found 20% to 50% increase in WALL%. A lower WALL% (11% to 22%) was measured by Wieland et al. [10]. Increased blood circulation and variations in vascularization might have increased the extent of WALL%, while the extent of circulatory impairment might reduce WALL% and result in a thickened APEX%. This suggestion would be in accordance with the study by Stauffer et al. [28], in which the thickening of the teat wall post-milking was mainly attributed to the increased diameter of the veins and not to the accumulation of extravascular fluid. Hamann et al. [6] suggested that the increased teat-skin blood flow was due to a “reactive hyperemia”, a tissue reaction to compensate for the mechanical impact during machine milking. On the other hand, decreased blood flow was explained by fluid accumulation at the aspect of the teat barrel interfering with the microcirculation in the teat skin [6].

The effect of CISTERN and CISTERN% was not well defined, although they had affected milking-induced changes in all monitored teat structures. In our study, the average CISTERN% was −34%, while Neijenhuis et al. [1] observed an average decrease of 45.8%. The accumulation of milk in the teat cistern during the milking interval can increase pressure, causing expansion of the teat canal diameter, most noticeably at the proximal end [3], and the significant effect of CISTERN size on CANAL% was noticeable in our results. The drop of intramammary pressure following reduced refill of the teat caused a loss in friction between the teat and the liner [25,29], and we could see that the wide CISTERN reacted by a high WALL% and the narrow CISTERN reacted by increased BARREL% and APEX%.

The highest range of APEX% was found for the dimension of the APEX before milking (thin APEX +3.86% vs. thick APEX −4.81%; *p* < 0.05). The teat end is slow to recover after milking and can take more than 8 h [1]. Moreover, we could see similarities for a high APEX% with results for BARREL and CISTERN. When the APEX% was in higher negative values (thick APEX, thick BARREL, and wide CISTERN), the BARREL% was over −10%. Even though a negative BARREL% seems to be a positive sign, Zwertvaegher et al. [17] hypothesized the existence of a critical threshold for the maximum reduction in teat barrel diameter, below which udder health is negatively affected. We observed a more favorable change of teat tissue for a medium APEX. However, from an udder health standpoint, Guarín and Ruegg [15] found an increased chance for mastitis by 20%, with each increase in the pre-milking diameter of APEX by 1 mm, and the authors attributed it to wider teat canals and larger orifices in wider teats.

A unique situation was observed for the CANAL when a short CANAL achieved a positive APEX%, while having the highest reduction in the BARREL% and the highest increase in the WALL%. For other structures, the results were the opposite. Vacuum-induced forces during milking will fracture the keratin layer and expand the tissue surrounding the teat canal [10]. Up to a certain level, this process seems to be a normal physiological response [7]. A short CANAL tends to have the highest milk flow [16], and the teat-end vacuum drop is increased with high milk flow rates [30]. Overmilking causes an increase in the teat surface temperature, thickening of the WALL, a decrease of the BARREL, and prolonging of the CANAL [5], and we observed these changes for short CANALs. The CANAL only recovers slowly to its pre-milking dimensions (>8 h) [1,3], and with these high changes for a short CANAL, teats with short canals might be in a state of near-constant remodeling [3]. Furthermore, the stretching of teat tissue causes micro fissures in the skin, which respond with increased keratin production [7]. Klein et al. [11] recommended long and narrow teat canals to improve udder health. Teat canal length in Holstein cows was reported to be 10.0 mm [1], 10.73 mm [31], or 14.24 mm [3] before milking. Studies showed that the average milking-induced changes in the teat canal length were 12% [1], 27% [31], or 11.1% [3]. These divergent outcomes could be explained by the application of different milking techniques, different milk flows, and, as our study supports, by differences in the morphological variation of the teat tissue of the experimental animals.

In our opinion, the relationship between milking-induced changes, and udder health and milkability is non-linear, and too low, as well as too high, of a change may negatively affect the teat. Based on our findings and the findings of the discussed studies, optimal teat tissue response to milking could be in the following range: 6 to 14% in LENGTH%; −4 to −8% in BARREL%; −35 to −45% in CISTERN%; 30 to 45% in WALL%; −2 to 1% in APEX%; and 10 to 20% in CANAL%. Nevertheless, milking machine effects on teats are influenced by various factors and are not well defined, and additional research is required to understand this relationship [15,32]. Results and findings of these studies could be used to create a detailed computer model of the teat and its reaction to various milking settings, which could be beneficial for the development of milking equipment and breeding for internal teat morphology traits. A better understanding of milking-induced changes in teat structures could also help optimize the milking settings for cow physiology and improve udder health. Optimizing the milking parlor setting for better adaptation to dairy physiology could reduce teat damage and slow morphological changes during production life [33,34]. Wieland et al. [10] concluded that milking cows with the same milking liner and identical machine settings may fail to accommodate the requirements of cows with different teat characteristics and possibly have a detrimental effect on the condition of teat tissue. The need for preventive protection of the primary defense mechanisms of the teats will increase as microbial resistance to antibiotics is expected to grow gradually.

## 5. Conclusions

Favorable milking-induced teat tissue changes were observed for teats of medium LENGTH, medium BARREL and APEX, with a medium CANAL, but wider CISTERN and thinner WALL. The way in which the teat is developed at the BARREL seems to be crucial, as 25 mm thick teats might consist of a wide CISTERN and thinner WALL or a narrow CISTERN with a thicker WALL. A worse tissue reaction to milking was found for a thin BARREL, narrow CISTERN, and thick WALL, as these dimensions were associated with short-term changes that were observed to have negative implications for milking efficiency or udder health by previous studies. The results of this study may help improve research in the area of milking-induced changes in teat morphology. Furthermore, our findings and the findings of other studies in this research area could help create a detailed computer model for the reaction to milking, which could be used in the development of milking equipment or breeding for internal teat morphology traits.

## Figures and Tables

**Table 1:** The effect of lactation number, lactation stage, and teat position on the least-square means ± SEM of milking-induced changes in teat structures.

Effect	Level	LENGTH% ^1^	BARREL% ^2^	CISTERN% ^3^	WALL% ^4^	APEX% ^5^	CANAL% ^6^
Lactation Number	1	12.69 ± 1.039 ^A^	−5.10 ± 0.622	−38.05 ± 1.865 ^A^	35.50 ± 2.187 ^A^	0.58 ± 0.578	17.66 ± 1.259 ^A^
	2+	9.32 ± 0.706 ^B^	−5.22 ± 0.422	−31.82 ± 1.267 ^B^	27.34 ± 1.486 ^B^	−0.56 ± 0.393	11.43 ± 0.855 ^B^
Lactation Stage	early	3.25 ± 1.037 ^A^	−6.29 ± 0.621	−42.65 ± 1.862 ^A^	40.68 ± 2.184 ^A^	−0.24 ± 0.578	20.70 ± 1.257 ^A^
	mid	14.28 ± 1.037 ^B^	−4.58 ± 0.621	−31.60 ± 1.862 ^B^	25.63 ± 2.184 ^B^	0.22 ± 0.578	10.64 ± 1.257 ^B^
	end	15.47 ± 1.037 ^B^	−4.61 ± 0.621	−30.56 ± 1.862 ^B^	27.95 ± 2.184 ^B^	0.05 ± 0.578	12.30 ± 1.257 ^B^
Teat Position	Front	8.06 ± 0.858 ^A^	−5.32 ± 0.513	−35.16 ± 1.539	27.96 ± 1.805 ^A^	0.67 ± 0.477 ^a^	14.20 ± 1.039
	Rear	13.94 ± 0.858 ^B^	−4.99 ± 0.513	−34.71 ± 1.539	34.88 ± 1.805 ^B^	−0.65 ± 0.477 ^b^	14.89 ± 1.039

^1^ Milking-induced change in teat length. ^2^ Milking-induced change in teat thickness at the barrel. ^3^ Milking-induced change in teat cistern width. ^4^ Milking-induced change in teat wall width. ^5^ Milking-induced change in teat thickness at the apex. ^6^ Milking-induced change in teat canal length. ^A,B,a,b^ Statistical significance *p* < 0.05.

**Table 2:** The effect of teat length, barrel thickness, and apex thickness on the least-square means ± SEM of milking-induced changes in teat structures.

Effect	Group (mm)	LENGTH% ^1^	BARREL% ^2^	CISTERN% ^3^	WALL% ^4^	APEX% ^5^	CANAL% ^6^
**LENGTH ^7^**	<43.5 Short	19.83 ± 1.163 ^A^	−3.75 ± 0.859 ^A^	−29.60 ± 2.467 ^A^	25.13 ± 2.937 ^A^	0.08 ± 0.785	12.22 ± 1.714 ^A^
	43.5–52.5 Medium	7.78 ± 1.081 ^B^	−5.42 ± 0.803	−39.18 ± 2.296 ^B^	37.57 ± 2.738 ^B^	0.37 ± 0.731	17.01 ± 1.597 ^B^
	>52.5 Long	1.63 ± 1.390 ^C^	−6.97 ± 0.991 ^B^	−37.67 ± 2.889	33.18 ± 3.416	−0.56 ± 0.914	14.96 ± 1.994
**BARREL ^8^**	<24.2 Thin	13.42 ± 1.312 ^A^	0.95 ± 0.675 ^A^	−24.86 ± 2.367 ^A^	26.46 ± 2.884 ^A^	3.55 ± 0.736 ^A^	11.86 ± 1.677 ^A^
	24.2–26.5 Medium	11.32 ± 1.252	−5.08 ± 0.642 ^B^	−36.92 ± 2.253 ^B^	31.65 ± 2.745	−0.48 ± 0.700 ^B^	13.92 ± 1.596
	>26.5 Thick	8.65 ± 1.270 ^B^	−10.82 ± 0.658 ^C^	−42.35 ± 2.303 ^B^	35.66 ± 2.810 ^B^	−2.79 ± 0.717 ^C^	17.58 ± 1.635 ^B^
**APEX ^9^**	<21 Thin	12.32 ± 1.242	−2.33 ± 0.774 ^A^	−29.83 ± 2.351 ^A^	28.95 ± 2.827	3.86 ± 0.703 ^A^	14.33 ± 1.632
	21–22.5 Medium	10.34 ± 1.182	−5.87 ± 0.734 ^B^	−37.81 ± 2.230 ^B^	33.50 ± 2.681	−1.51 ± 0.667 ^B^	14.94 ± 1.548
	>22.5 Thick	9.62 ± 1.563	−9.48 ± 0.943 ^C^	−39.67 ± 2.901 ^B^	32.36 ± 3.468	−4.81 ± 0.857 ^C^	14.24 ± 2.003

^1^ Milking-induced change in teat length. ^2^ Milking-induced change in teat thickness at the barrel. ^3^ Milking-induced change in teat cistern width. ^4^ Milking-induced change in teat wall width. ^5^ Milking-induced change in teat thickness at the apex. ^6^ Milking-induced change in teat canal length. ^7^ teat length. ^8^ Teat thickness at the barrel. ^9^ Teat thickness at the apex. ^A,B,C^ Statistical significance *p* < 0.05.

**Table 3:** The effect of teat cistern width, wall thickness, and canal length on the least-square means ± SEM of milking-induced changes in teat structures.

Effect	Group (mm)	LENGTH% ^1^	BARREL% ^2^	CISTERN% ^3^	WALL% ^4^	APEX% ^5^	CANAL% ^6^
**CISTERN ^7^**	<11 Narrow	14.31 ± 1.426 ^A^	0.27 ± 0.716 ^A^	−17.06 ± 2.472 ^A^	13.49 ± 2.921 ^A^	3.10 ± 0.797 ^A^	6.48 ± 1.809 ^A^
	11–14.5 Medium	10.81 ± 1.045	−4.57 ± 0.516 ^B^	−38.24 ± 1.887 ^B^	33.39 ± 2.222 ^B^	−0.15 ± 0.603 ^B^	15.00 ± 1.412 ^B^
	>14.5 Wide	8.78 ± 1.351 ^B^	−10.45 ± 0.673 ^C^	−42.79 ± 2.384 ^B^	41.75 ± 2.813 ^C^	−2.10 ± 0.766 ^C^	19.99 ± 1.758 ^C^
**WALL ^8^**	<5.7 Thin	9.77 ± 1.350	−6.77 ± 0.831 ^A^	−40.21 ± 2.429 ^A^	50.59 ± 2.498 ^A^	−0.79 ± 0.785 ^A^	19.77 ± 1.734 ^A^
	5.7 to 7 Medium	11.26 ± 1.122	−5.96 ± 0.710 ^A^	−39.18 ± 2.056 ^A^	32.71 ± 2.105 ^B^	−0.56 ± 0.668 ^A^	15.59 ± 1.492 ^B^
	>7 Thick	11.75 ± 1.263	−2.73 ± 0.787 ^B^	−24.89 ± 2.291 ^B^	13.08 ± 2.352 ^C^	1.44 ± 0.742 ^B^	8.67 ± 1.647 ^C^
**CANAL ^9^**	<11.8 Short	8.67 ± 1.900	−8.76 ± 1.167 ^A^	−45.12 ± 3.461 ^A^	46.78 ± 3.951 ^A^	1.15 ± 1.094	35.41 ± 2.006 ^A^
	11.8–14 Medium	11.28 ± 1.044	−5.30 ± 0.731 ^B^	−35.77 ± 2.045 ^B^	32.17 ± 2.316 ^B^	−0.03 ± 0.656	14.73 ± 1.216 ^B^
	>14 Long	11.81 ± 1.423	−2.69 ± 0.924 ^C^	−27.04 ± 2.673 ^C^	20.59 ± 3.041 ^C^	−0.59 ± 0.850	1.62 ± 1.566 ^C^

^1^ Milking-induced change in teat length. ^2^ Milking-induced change in teat thickness at the barrel. ^3^ Milking-induced change in teat cistern width. ^4^ Milking-induced change in teat wall width. ^5^ Milking-induced change in teat thickness at the apex. ^6^ Milking-induced change in teat canal length. ^7^ Width of teat cistern. ^8^ Teat thickness at the barrel. ^9^ teat canal length. ^A,B,C^ Statistical significance *p* < 0.05.

## Data Availability

The data presented in this study are available in a publicly accessible repository, Mendeley Data, at https://doi.org/10.17632/tstnn6m75z.2 [20] (accessed on 13 March 2023).

## Supplementary Materials

The following supporting information can be downloaded at: https://doi.org/10.17632/tstnn6m75z.2 [20], Table S1. The significance of effects in the model equation focused on the evaluation of teat length on milking-induced changes of teat structures; Table S2. The significance of effects in the model equation focused on the evaluation of barrel thickness on milking-induced changes of teat structures; Table S3. The significance of effects in the model equation focused on the evaluation of cistern width on milking-induced changes of teat structures; Table S4. The significance of effects in the model equation focused on the evaluation of wall thickness on milking-induced changes of teat structures; Table S5. The significance of effects in the model equation focused on the evaluation of apex thickness on milking-induced changes of teat structures; Table S6. The significance of effects in the model equation focused on the evaluation of canal length on milking-induced changes of teat structures, Supplementary results, Supplementary discussion.
